# The relationship between hand injuries due to occupational accidents and Vitamin D deficiency

**DOI:** 10.4314/ahs.v25i2.24

**Published:** 2025-06

**Authors:** Ali Sağlık, Tufan Akın Giray, Tarık Ocak

**Affiliations:** Istinye Universitesi, Emergency Medicine, Istanbul, İstanbul

**Keywords:** Hand injuries, hand fractures, occupational accidents, vitamin D

## Abstract

**Background:**

Bone fractures due to occupational accidents cause labor and cost losses in the world. Vitamin D deficiency is common in the world including industrialized countries. This study aimed to investigate the relationship between fracture and vitamin D levels in patients with wrist fractures due to occupational accidents.

**Methods:**

This study was conducted in a study group consisting of patients who were admitted to the emergency department of a university hospital due to occupational accidents and were thought to have a fracture of the hand or wrist. Blood samples were analyzed biochemically and vitamin D levels were obtained.

**Results:**

Vitamin D3 level was 16.3 ng/mL in patients with fractures and 21 ng/mL in patients without fractures, which was statistically significant (p<0.05). Vitamin D deficiency was found in %60.2 of patients with fractures, while vitamin D deficiency was found in %50.2 of patients with fractures and there was no statistical difference (p>0.112). Vitamin D deficiency was found in %39.8 of patients with hand injury without fracture, whereas vitamin D deficiency was not detected in %33 of patients with hand injury and the comparison was statistically significant (p<0.05)

**Conclusion:**

There is insufficient data in the literature on the relationship between bone fractures and vitamin D levels and the vitamin D levels required to prevent bone fractures. In this study, a statistically significant difference was found between the vitamin D levels of patients with fractures and those of patients without fractures. This study is beneficial for the literature in this respect, but extensive studies on the relationship between vitamin D and fractures are needed.

## Introduction

The importance of occupational health and safety is rapidly increasing both in the world and in our country. Bone fractures are an important type of injuries caused by occupational accidents. Bone fractures occur when the human body is exposed to a high level of force. Unfortunately, it leads to labor and cost losses all over the world and in our country^1^. Vitamin D maintains PTH levels at physiologically healthy levels, increases osteoblastic activity, and promotes bone mineralization, which in turn significantly reduces the risk of falls and fractures^2^. There is evidence that patients with vitamin 25(OH)D3 levels >30 ng/mL have a lower risk of fracture^3^. Vitamin D deficiency is now recognized as a global epidemic. Vitamin D deficiency and 20.7% of vitamin D insufficiency were found^4^.

This study aimed to obtain sociodemographic data on patients with wrist fractures due to occupational accidents and to investigate the relationship between characteristics and vitamin D levels.

## Methods

This prospective study was conducted in the emergency department of a university hospital in the metropolitan area of Istanbul with approximately 43000 emergency patient admissions per year. Informed consent was obtained from all participants, and the study was monitored by the local ethical committee. The study group consisted of patients who presented to the emergency department for the first time as an occupational accident during the 3-year (2019-2021) study period and were thought to have a fracture of the hand and wrist by the emergency physician. Patients with multiple traumas, patients who refused to participate in the study, patients with sharps injuries, amputations, and burns, and patients younger than 16 years of age were excluded.

Many similar studies have been conducted to define vitamin D deficiency and insufficiency and to determine the normal range of 25(OH)D3 vitamin levels^3^. In the light of these studies, vitamin D deficiency was accepted if the 25(OH)D3 vitamin level was lower than 20 ng/mL, vitamin D insufficiency if the level was between 21 and 29 ng/mL, and adequate if the level was higher than 30 ng/mL^3^. Blood samples from the patients were transported to the hospital laboratory under appropriate conditions without waiting and the results were obtained by luminescence immunoassay on an Abbott Architect Autoanalyzer (GMI, Ramsey, USA) (reference values 30-80 ng/mL, deficiency <20 ng/mL).

## Statistical Analysis

The study data were analyzed with MedCalc® Statistical Software version 22.006 (MedCalc Software Ltd, Ostend, Belgium; https://www.medcalc.org; 2023). Numeric data were expressed as a median and interquartile ratio (IQR) and frequent variables as rates. Comparison of two independent groups with numeric data was performed by Mann Whitney U test, and the Chi-square test was used for categorical data. The normality analysis was conducted by the Kolmogorov-Smirnov test.

All the hypotheses were constructed as two-tailed and an alpha critical value of 0.05 was accepted as significant.

## Results

**Figure 1:**
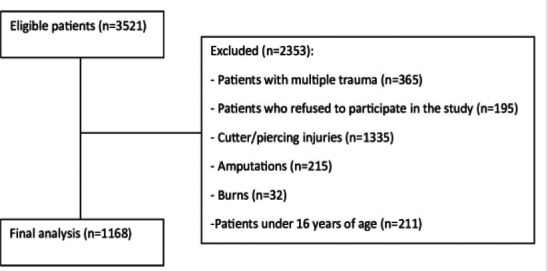
Patient flow chart

The mean age of the 1168 patients who met the inclusion criteria was 38 years (min 16-max 77), 108 of them were female and 1060 of them were male, out of 3521 occupational accident cases admitted to our emergency department with hand injuries. Among the patients, 341 (29.2%) were smokers and the rest (N:827) were non-smokers. Among the patients who presented with hand injuries after occupational accidents, the most common type of occupational accident was cutting/piercing type injuries (N:1335). (Figure 1) Among these patients, the number of metal-machinery workers was 565, woodworkers 233, service personnel 125, textile workers 108, construction workers 74, and other workers 63. When the mechanisms of injury were analyzed, crush-type injuries were found to be the most common (N:883). (Table 1)

**Table 1:** Demographic variables of study patients

Variable	N (%)
Age,	
Median (IQR)	38 (29 to 45)
Min-max	16-77
Gender	
Female	108 (9.2)
Male	1060 (90.8)
Smoking	
Yes	341 (29.2)
No	827 (70.8)
Sectors of industry	
Metal-machinery	565 (48.4)
Wood-furniture	233 (19.9)
Services	125 (10.7)
Textile	108 (9.2)
Construction	74 (6.3)
Other	63 (5.4)
Mechanisms of injury	
Crush Injuries	883 (75.6)
Falls	198 (17.0)
Sprains	39 (3.3)
Involving more than one mechanism	48 (4.1)

Abbreviations: IQR: interquartile range

In this study, the median 25(OH)D3 levels of women were 20.9 (IQR: 11.4-25.5) ng/mL and 18.7 (IQR: 11.5-25.5) ng/mL in men. 25(OH)D3 levels of the study patients are given in Table 2. The mean 25(OH)D3 levels of all patients was 18.7 (IQR: 11.5-25.5) and 25(OH)D3 insufficiency was found in 86.9% (N:1015) of the patients, while 25(OH)D3 deficiency was found in 54.3% (N:634) of all patients.

**Table 2:** 25(OH)D3 levels of study patients

Variable	N (%)
Vitamin D3, median (IQR)	18.7 (11.5 to 25.5)
Gender, median (IQR)	
Female	20.9 (11.4-25.5)
Male	18.7 (11.5-25.5)
Vit D insufficiency*	
Yes	1015 (86.9)
No	153 (13.1)
Vit D deficiency**	
Yes	634 (54.3)
No	534 (45.7)

Abbreviations: IQR: interquartile range

* Vitamin D levels <30 ng/mL

** Vitamin D levels <20 ng/mL

Fractures were detected in 693 patients, of which 109 patients had forearm fractures, 12 patients had carpal bone fractures, 97 patients had metacarpal fractures, 422 patients had phalanx fractures and the remaining 53 patients had multiple fractures. Right-sided injuries (N:700) were frequently observed in the admitted patients, while left-sided injuries were observed in 450 patients and bilateral injuries were observed in 18 patients. When finger injuries were analyzed, it was found that the second finger was the most common injury followed by the third finger. In cases with more than one finger injury, the 3^rd^ and 4th finger injuries were frequently associated. While conservative treatment was generally applied to the injured patients, 43.1% (N:503) of the applicants underwent surgical procedures. (Table 3)

**Table 3:** Descriptive data of fractures identified in the study patients

Variable	N (%)
Fracture	
Yes	693 (59.3)
No	475 (40.7)
Fractured Bone	
Forearm	109 (15.7)
Carpal bones	12 (1.7)
Metacarpus	97 (14.1)
Phalanx	422 (60.9)
More than one site	53 (7.6)
Injury Site	
Right Site	700 (60)
Left Site	450 (38.5)
Bilateral	18(1.5)
Fracture Phalanx	
1^st^ Phalanx	67 (11.7)
2^nd^ Phalanx	118 (20.6)
3^rd^ Phalanx	113 (19.8)
4^th^ Phalanx	82 (14.3)
5^th^ Phalanx	87(15.2)
1^st^ and 2^nd^ Phalanx	5 (0.9)
1^st^ and 3^rd^ Phalanx	3 (0.5)
2^nd^ and 3^rd^ Phalanx	36 (6.3)
3^nd^ and 4^th^ Phalanx	32 (5.6)
4^th^ and 5^th^ Phalanx	17 (3)
2^nd^, 3^rd^ and 4^th^ Phalanx	3 (0.5)
3^rd^, 4^th^ and 5^th^ Phalanx	9 (1.6)
Treatment	
Conservative	665 (56.9)
Surgical treatment	503 (43.1)

**Figure 2:**
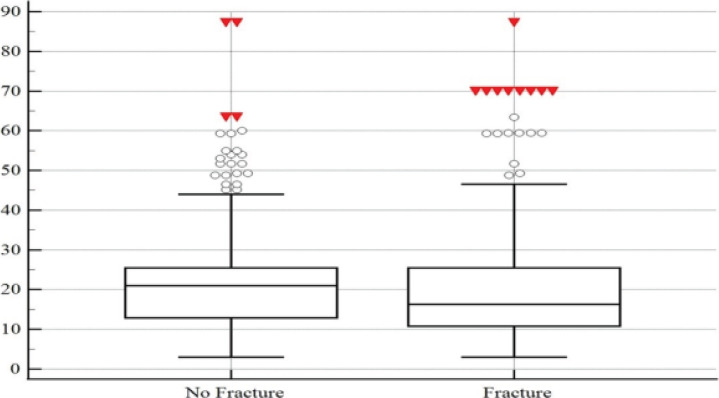
Vitamin D3 levels (ng/mL) of study patients with and without fracture

Vitamin D3 level was 16.3 ng/mL in patients with fractures and 21 ng/mL in patients without fractures, which was statistically significant (p<0.05). Vitamin D3 deficiency was found in 60.2% of the patients with fractures, while vitamin D3 deficiency was found in 50.2% of patients with no fractures and no statistically significant differences were found (p>0.112). Vitamin D3 insufficiency was found in 39.8% of patients with hand injury without fracture, whereas 33% did not have vitamin D3 deficiency and the comparison was significant (p<0.05). (Table 4) (Figure 2)

**Table 4:** Comparison of Vitamin D3 levels of study patients with and without fractures

Variable	Patients with Fractures	Patients without Fractures	P Value
Vitamin D3 levels (ng/mL)	16.3 (10.8 to 25.5)	21 (20.1 to 21.7)	<0,001
Vitamin D3 Insufficiency (<30 ng/mL), N (%)			
No	82 (53.6)	71 (46.4)	0.112
Yes	611 (60.2)	404 (39.8)	
Vit D3 Defciency (<20 ng/mL), N (%)			
No	268 (50.2)	266 (49.8)	
Yes	425 (67)	209 (33)	

## Discussion

The hand is recognized as the most utilized anatomical structure in the body, primarily due to its multifaceted capabilities, yet it is simultaneously highly prone to injuries. This vulnerability stems from its intricate anatomical composition, which includes 20 muscles, 27 bones, an extensive network of tendons, blood vessels, and nerves. A predominant factor contributing to hand injuries is incidents occurring in occupational and sports settings^5^,^6^. Data from a United States study indicates that 35.1% of workers experience job-related injuries annually, with approximately one million of these being hand-related injuries^5^,^7^-^9^. The frequency of occupational traumatic hand injuries varies across different industries, with reported rates ranging from 4 to 11 injuries per 100 workers per year^10^. Notably, hand and finger injuries are the most prevalent type of occupational injury recorded in emergency departments, constituting about 15% of total injuries. These injuries can range from minor lacerations and sprains to severe conditions resulting in disability or amputation^11^,^12^.

The most frequently reported injuries treated in emergency settings are traumas involving the upper extremities, particularly the fingers and hands^12^. Among these, hand fractures are the most common, occurring three times more often than distal radius fractures; globally, fractures of the hand, specifically metacarpal and phalanx fractures are the most widely occurring fracture types^13^. Work-related fractures have a notable incidence in the phalanges (15%), followed by foot bones (9%) and carpal bones (8%)^12^,^14^. In our analysis, fractures were observed in 60.9% of cases within the phalanges, 15.7% in the forearm, 14.1% in the metacarpals, and 1.7% in the carpal bone, with 7.6% exhibiting fractures at multiple sites. Furthermore, research in Wasit Governorate corroborated the phalanges as the predominant fracture site^15^. Metacarpal fractures represent approximately 18-44% of all hand fractures recorded in the literature^16^. Studies conducted by Tayal et al. and Ghiya et al. revealed that 32% and 26% of hand fractures were attributed to phalanx injuries, while metacarpal injuries accounted for 68% and 74%, respectively^11^,^17^. These findings underscore the variability in fracture incidence depending on the research context.

Vitamin D plays a critical role in maintaining calcium homeostasis and regulating skeletal health, primarily synthesized through skin exposure to sunlight or through dietary intake^4^. The metabolite 25(OH)D3 is employed as a standard marker for evaluating an individual's vitamin D status, with established thresholds categorizing levels <20 ng/mL as deficient, 21-29 ng/mL as insufficient, and >30 ng/mL as adequate^3^. Canadian studies have reported that 30-50% of individuals exhibit vitamin D deficiency, and similarly, data from 2001-2006 indicated that 33% of the US population was affected by this condition^18^,^19^,^20^. Moreover, investigations into populations in India, Africa, Australia, Brazil, and Saudi Arabia have found significant vitamin D deficiency rates across adults and children alike^3^,^21^-^25^. A systematic review encompassing 71 global studies identified occupational exposure as a significant determinant of vitamin D status, highlighting that individuals working indoors have a heightened risk of developing hypovitaminosis D compared to their outdoor counterparts^26^. Further research demonstrated markedly lower serum vitamin D levels among shift workers in contrast to non-shift workers, with a particular study in Italy reporting levels of 16.3 ng/mL for shift workers versus 21.9 ng/mL for those working standard hours^27^,^28^. In our analysis, despite the lack of regional studies linking vitamin D levels to occupational hand fractures, Uçar et al. reported that among adults presenting with non-specific complaints in Ankara, a significant proportion exhibited vitamin D deficiency (51.8%) and insufficiency (20.7%)^4^. Vitamin D levels are influenced by geographical location, lifestyle choices, dietary preferences, gender, and genetic predispositions. Collectively, this data indicates a widespread occurrence of vitamin D deficiency and insufficiency across diverse populations and age groups.

Globally, it is estimated that about one billion individuals experience insufficient or deficient levels of vitamin D^24^. In our study population, the median 25(OH)D3 levels were documented at 20.9 ng/mL for women and 18.7 ng/mL for men, with an overall mean of 18.7 ng/mL. A concerning 86.9% and 54.3% of participants demonstrated vitamin D deficiency and insufficiency, respectively, findings that are consistent with existing literature. An analysis of vitamin D levels in those with fractures revealed an average of 16.3 ng/mL compared to 21 ng/mL in non-fractured counterparts, a difference deemed statistically significant.

Vitamin D deficiency predisposes individuals to secondary hyperparathyroidism, prompting increased bone resorption, thereby amplifying fracture risk^4^. In adults, insufficient 25(OH)D3 levels can lead to impaired mineralization of the collagen matrix, weakening structural integrity and elevating the fracture risk. However, the requisite vitamin D concentration for fracture prevention remains indeterminate^29^. Research evaluating the impact of vitamin D supplementation on fracture susceptibility reveals mixed outcomes, with some reports indicating significant reductions in fracture incidence, while others do not. Notably, a meta-analysis encompassing over 30,000 subjects found that vitamin D supplementation at doses ≥792 IU/day resulted in a substantial reduction in fracture risk—30% for hip fractures and 14% for non-vertebral fractures^30^.

Additionally, literature indicates that nearly half of patients treated for hand injuries after occupational accidents are smokers^1^,^5^,^7^. Bhatti et al. highlighted that smoking among workers may contribute to diminished focus and increase susceptibility to injuries on the job^5^. Our own findings indicated that 29.2% of patients in our study were smokers—a considerably lower rate compared to other reports—potentially attributable to stringent anti-smoking regulations in our country, which have contributed to a decrease in smoking prevalence among younger populations.

## Conclusions

Occupational accidents remain a major concern in developing countries and have serious public health consequences. Vitamin D deficiency is a common health problem worldwide, but there is a paucity of data in the literature on its association with bone fractures and the specific levels of vitamin D needed to prevent such fractures. Although additional and more extensive research is needed to better understand the link between vitamin D deficiency and fractures, our study provides valuable information in this regard.

The findings of our study emphasize the critical role of vitamin D supplementation, a balanced diet, regular physical activity and adequate sunlight exposure in promoting bone health. To address the high incidence of occupational fractures, we recommend regular screening of workers in high-risk sectors for vitamin D deficiency. Furthermore, occupational health policies should include targeted interventions such as vitamin D supplementation programs, educational campaigns on bone health and workplace strategies to reduce fracture risk. These measures can significantly contribute to reducing both the prevalence of vitamin D deficiency and the incidence of fractures among workers.

## Limitations

When the sample size and the nature of the study are examined, the power analysis shows that the number of participants is sufficient and the study has the necessary power to detect the predetermined effect size. However, since the participants of our study included only cases in Istanbul province of Turkey, we believe that further studies with a large patient population are needed to determine the relationship between vitamin D levels and multifactorial causes such as ethnic, geographical, dietary habits, duration of exposure to sunlight, comorbidities, medications and treatments.
